# Giant Cavernous Hepatic Hemangioma Masking a Separate Pleural Empyema: A Diagnostic Pitfall and Anchoring Bias

**DOI:** 10.7759/cureus.111256

**Published:** 2026-06-21

**Authors:** Lina Ataya, John P Martinez Ponce, Sandeep K Bhangu, Aceil Shamieh

**Affiliations:** 1 Internal Medicine, Henry Ford Genesys Hospital, Grand Blanc, USA; 2 Internal Medicine, Henry Ford Health System, Grand Blanc, USA; 3 Internal Medicine, Michigan State University, East Lansing, USA

**Keywords:** anchoring bias, cavernous hepatic hemangioma, diagnostic pitfall, empyema, pleural effusion

## Abstract

Giant cavernous hepatic hemangiomas may occasionally demonstrate atypical imaging characteristics that mimic malignant or infectious hepatic lesions, creating diagnostic uncertainty and increasing the risk of anchoring bias. We report the case of a 41-year-old male who presented with fever, pleuritic chest pain, leukocytosis, and a large right pleural effusion following CT-guided lung biopsy. Imaging revealed a large hepatic lesion with atypical radiographic features concerning for malignancy or disseminated infection, along with pulmonary inflammatory changes and empyema. Thoracentesis yielded pleural fluid concerning for empyema. Given the constellation of findings, the hepatic lesion initially dominated diagnostic reasoning and raised concern for a unifying metastatic or infectious process. However, liver biopsy ultimately confirmed cavernous hepatic hemangioma, while pulmonary biopsy demonstrated inflammatory changes without evidence of malignancy. Histopathologic evaluation established that the hepatic lesion was incidental and unrelated to the patient’s acute illness, whereas the empyema was most likely secondary to the recent thoracic intervention. This case highlights the importance of avoiding anchoring bias and recognizing concurrent but unrelated disease processes when evaluating complex clinical presentations.

## Introduction

Cavernous hepatic hemangioma is the most common benign hepatic tumor and is frequently discovered incidentally during abdominal imaging studies [1]. Histologically, these lesions are composed of large vascular channels lined by endothelial cells and are typically slow-growing and clinically silent. Most lesions are small and asymptomatic; however, giant hemangiomas (>10 cm) may demonstrate atypical radiographic characteristics that overlap with malignant or infectious hepatic lesions, often creating diagnostic uncertainty and necessitating further evaluation [2,3]. In some cases, giant hemangiomas may exhibit heterogeneous enhancement patterns, internal fibrosis, thrombosis, or central scarring, further complicating radiographic interpretation and increasing concern for hepatic malignancy [4,5].

Although hepatic hemangiomas are generally benign and require no intervention, symptomatic giant lesions may present with abdominal pain, compression of adjacent structures, consumptive coagulopathy, or spontaneous hemorrhage [6]. The broad differential diagnosis for atypical hepatic lesions includes focal nodular hyperplasia, hepatic adenoma, fibrolamellar hepatocellular carcinoma, metastatic disease, and hepatic abscesses [2,3]. Consequently, tissue sampling or advanced imaging modalities are occasionally required to establish a definitive diagnosis.

In patients presenting with systemic inflammatory symptoms, the coexistence of a large hepatic lesion may lead clinicians toward a unifying diagnosis, increasing the risk of diagnostic anchoring bias. Anchoring bias occurs when clinicians rely excessively on an initial prominent finding while insufficiently adjusting subsequent clinical reasoning despite evolving information [7,8]. This cognitive error has been recognized as an important contributor to delayed diagnosis and diagnostic inaccuracies in complex medical presentations [8].

Empyema, by contrast, is most commonly a complication of pneumonia but may also develop following thoracic trauma, invasive pulmonary procedures, or secondary infection of pleural fluid collections [4,9]. Post-procedural empyema following CT-guided lung biopsy is uncommon but represents a recognized complication associated with pleural contamination and disruption of pulmonary parenchyma [10]. This case illustrates how a prominent but incidental imaging finding can influence diagnostic reasoning and contribute to anchoring bias. Reporting such cases may increase awareness of cognitive diagnostic errors and reinforce the need to continually reassess differential diagnoses when evaluating complex clinical presentations.

## Case presentation

A 41-year-old male with a medical history significant for hypertension and a 17-pack-year smoking history presented to the emergency department in October 2025 with fever and pleuritic chest pain. The patient had quit cigarette smoking three years prior but continued nicotine vaping. History was obtained from both the patient’s wife and chart review.

One month prior to presentation, the patient experienced a syncopal episode resulting in nasal and rib fractures. Subsequent outpatient evaluation revealed iron deficiency anemia requiring intravenous iron therapy. Additional workup identified a right lung mass, prompting PET imaging and CT-guided lung biopsy one day prior to admission. The chest radiograph demonstrated a moderate right pleural effusion with associated right basilar airspace opacity concerning for parapneumonic effusion or empyema (Figure 1). A CT chest further revealed a large right-sided pleural effusion with adjacent right lower lobe consolidation, pleural-based inflammatory changes, patchy ground-glass opacities, and a small right pneumothorax following a CT-guided lung biopsy (Figure 2). Following the biopsy, the patient developed a fever up to 102.3°F, pleuritic chest pain, a nonproductive cough, and hemoptysis.

**Figure 1:**
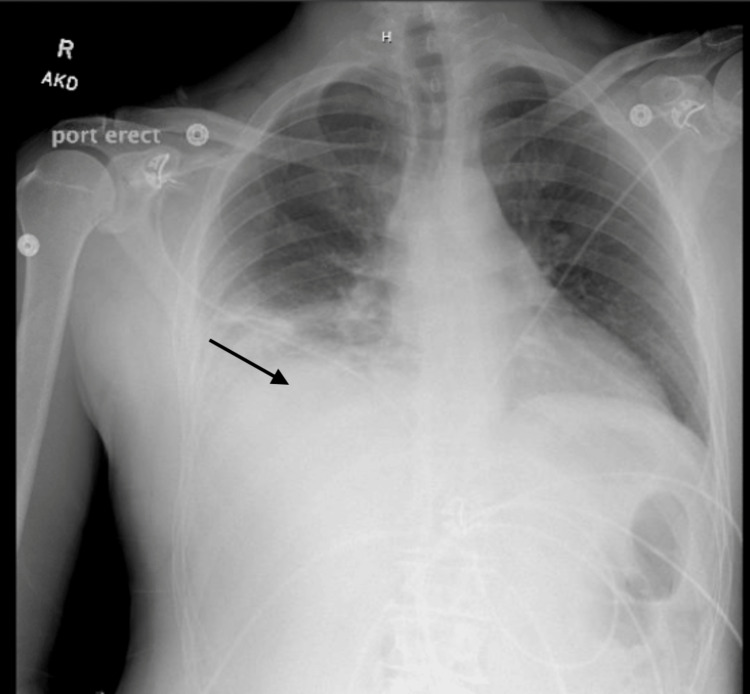
Portable chest radiograph demonstrating a moderate right pleural effusion with associated right basilar airspace opacity concerning for parapneumonic effusion or empyema.

**Figure 2:**
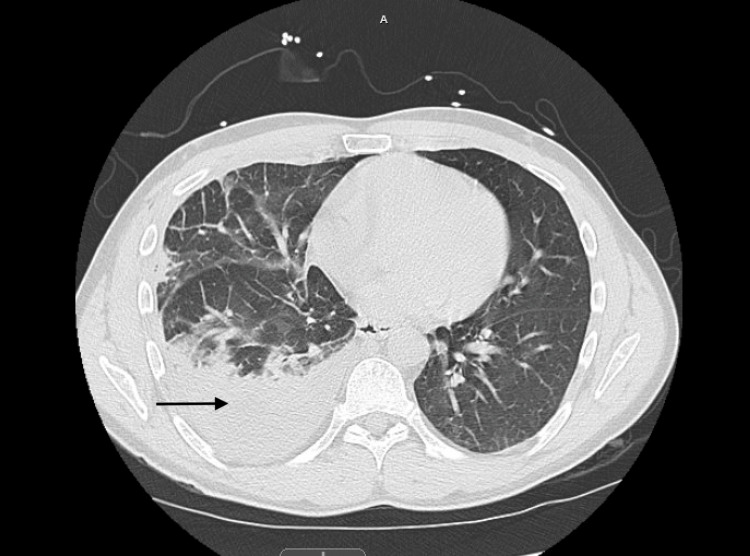
Axial CT chest image demonstrating a large right-sided pleural effusion with adjacent right lower lobe consolidation and pleural-based inflammatory changes following CT-guided lung biopsy.

On presentation, vital signs demonstrated mild tachycardia with a heart rate of 102 beats/min, blood pressure of 117/74 mmHg, respiratory rate of 20/min, temperature of 99.9°F, and oxygen saturation of 98% on a 2 L nasal cannula. Laboratory studies demonstrated leukocytosis and mild anemia (Table 1).

**Table 1:** Laboratory findings on presentation.

Laboratory Parameter	Result	Reference Range	Unit
White blood cell count	13.6	4.5–11.00	K/µL
Hemoglobin	11.6	12.0–17.3	g/dL

Following admission, a repeat CT of the chest demonstrated a large right-sided pleural effusion with associated compressive atelectatic and consolidative changes, a 4.3 × 6.3 cm right upper lobe pleural-based non-calcified soft-tissue density opacity, patchy ground-glass interstitial and alveolar opacities within the aerated right lung, and a small right apical pneumothorax.

Thoracentesis yielded serosanguinous pleural fluid. Pleural fluid analysis (Table 2) demonstrated an exudative effusion with marked neutrophilic predominance, elevated LDH, and markedly low glucose, consistent with pleural empyema in the appropriate clinical and radiographic context.

**Table 2:** Pleural Fluid Analysis.

Parameter	Result	Reference Range/Interpretation	Unit
Appearance	Serosanguinous	Clear, straw-colored	-
Red blood cell count	46,000	<1,000	cells/cmm
White blood cell count	14,000	<1,000	cells/cmm
Neutrophils	88	<25	%
Lymphocytes	5	Variable	%
Mononuclear cells	7	Variable	%
Protein	3.6	-	g/dL
Pleural fluid/serum protein ratio	>0.5	>0.5 favors exudate	-
LDH	1,857	>128 favors exudate	U/L
Glucose	<5	<60 mg/dL prompts consideration of empyema, tuberculosis, or malignant effusion	mg/dL
Gram stain	Moderate WBCs identified	No WBCs or organisms identified	-
Culture	Negative	Negative	-

Abdominal ultrasound revealed multiple hepatic lesions, including a dominant 10.2 cm lesion in the right hepatic lobe without internal vascularity. Magnetic resonance imaging (MRI) of the abdomen demonstrated a 10.3 × 10.1 × 9.1 cm lobulated mass involving hepatic segments V and VII. The lesion exhibited mixed T2 signal intensity with a thick peripheral rind of intermediate-to-hyperintense T2 signal and a central T2-hyperintense cord suggestive of a central scar (Figures 3, 4). Post-contrast images demonstrated peripheral rim enhancement with progressive enhancement of the peripheral rind and persistent non-enhancement of the central portion. Differential considerations included focal nodular hyperplasia, fibrolamellar carcinoma, metastatic disease, hepatic adenoma, and atypical hemangioma. Additional smaller hepatic lesions measuring up to 1.3 cm were also identified.

**Figure 3:**
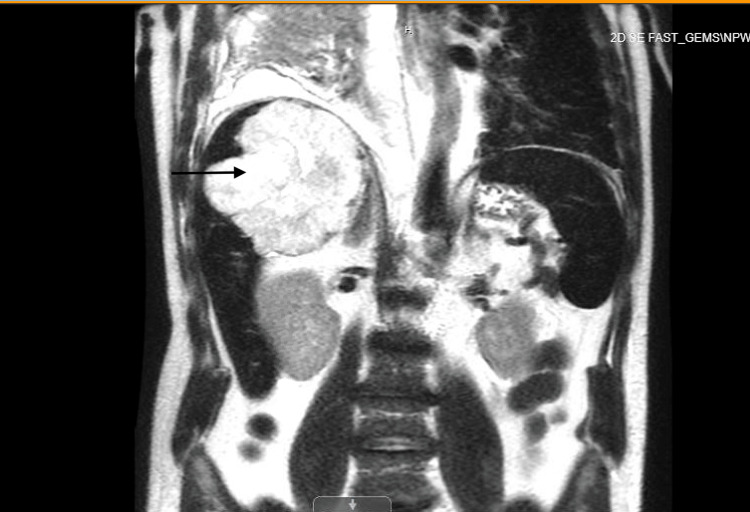
Coronal MRI abdomen demonstrating a large heterogeneous lesion involving the right hepatic lobe.

**Figure 4:**
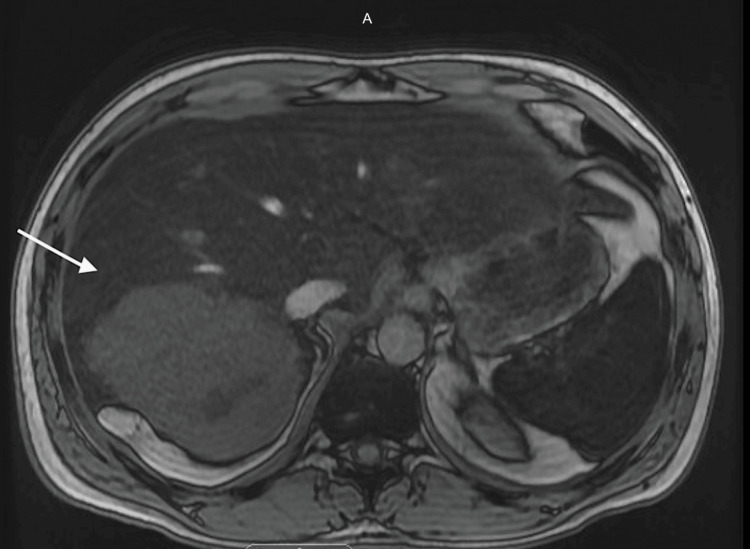
Axial MRI abdomen demonstrating a giant right hepatic lobe lesion measuring approximately 10 cm, later confirmed on biopsy as a cavernous hepatic hemangioma.

The liver lesion lacked typical imaging characteristics. Given the presence of a known lung mass and concern for possible metastatic disease, malignancy could not be confidently excluded based on imaging alone. Therefore, an image-guided liver biopsy was pursued to establish a definitive diagnosis despite the recognized bleeding risk associated with the biopsy of suspected hepatic hemangiomas.

Subsequent liver biopsy demonstrated cavernous hepatic hemangioma, while pulmonary biopsy revealed inflammatory changes without evidence of malignancy. The pleural empyema was ultimately determined to represent a separate thoracic process, likely related to the recent lung biopsy rather than the hepatic lesion itself.

The patient was treated for pleural empyema with intravenous vancomycin and piperacillin-tazobactam. A pigtail catheter was placed, and intrapleural tissue plasminogen activator (tPA) 10 mg and dornase alfa (DNase) 5 mg were administered twice daily for three days (total of six doses each). Repeat CT imaging demonstrated significant improvement of the pleural effusion, and no surgical intervention, including video-assisted thoracoscopic surgery (VATS) or decortication, was required. The patient was discharged home in stable condition with instructions to follow up at a tertiary care center for evaluation by the hepatobiliary surgery service regarding the atypical large symptomatic hepatic lesion.

## Discussion

Giant hepatic hemangiomas may present a substantial diagnostic challenge because atypical imaging characteristics can mimic malignant or infectious hepatic lesions [1-3]. While classic hemangiomas demonstrate peripheral nodular enhancement with progressive centripetal fill-in on contrast imaging, giant lesions may instead appear heterogeneous because of fibrosis, thrombosis, necrosis, or cystic degeneration [3,5]. These atypical radiographic features may closely resemble metastatic disease, hepatocellular carcinoma, hepatic abscess, or inflammatory lesions, thereby complicating diagnostic interpretation and management decisions [2,5].

In the present case, the coexistence of systemic inflammatory symptoms and a large hepatic mass initially raised concern for a unifying diagnosis involving metastatic disease or transdiaphragmatic infectious spread. The presence of fever, leukocytosis, pleural effusion, and multiple hepatic lesions further amplified concern for disseminated malignancy or multifocal infection. Similar diagnostic dilemmas have been described in prior reports where giant hemangiomas mimicked aggressive hepatic neoplasms or infectious processes because of atypical enhancement patterns and associated inflammatory findings [5,6].

However, histopathologic evaluation confirmed the liver lesion to be a benign cavernous hemangioma, effectively excluding it as the source of infection or systemic inflammation. The patient’s empyema was instead determined to be a separate pleural process likely associated with recent thoracic intervention. Empyema is commonly associated with pneumonia but may also occur following thoracic procedures, trauma, or secondary infection of pleural collections [4,9]. CT-guided transthoracic lung biopsy, although generally considered safe, carries recognized risks, including pneumothorax, hemorrhage, hemothorax, and, less commonly, infectious complications such as empyema [10,11]. In this patient, the temporal association between lung biopsy and the onset of fever, pleuritic chest pain, and worsening pleural effusion supported a post-procedural thoracic infectious process.

This case also highlights the importance of recognizing diagnostic anchoring bias, in which an early prominent finding disproportionately influences subsequent clinical reasoning [7]. The large hepatic lesion initially dominated the diagnostic evaluation and raised suspicion for a unifying pathology. Anchoring bias may become particularly problematic when imaging abnormalities appear visually striking or when clinicians attempt to consolidate multiple symptoms into a single diagnosis [8,12]. In complex clinical presentations, premature diagnostic closure may delay recognition of concurrent but unrelated disease processes.

Only through histopathologic confirmation and continued reassessment was it recognized that the patient had two concurrent but unrelated disease processes. This case underscores the importance of maintaining a broad differential diagnosis and continually re-evaluating diagnostic assumptions as additional clinical information becomes available. Awareness of cognitive biases is especially critical in patients with overlapping inflammatory features and atypical imaging findings, where reliance on an initial diagnostic impression may obscure the true etiology of acute illness [8,12].

## Conclusions

This case demonstrates the diagnostic pitfall of attributing systemic inflammatory illness to a striking but incidental imaging abnormality. Although giant cavernous hepatic hemangiomas may appear radiographically concerning, they are generally benign and unrelated to systemic infection. Careful diagnostic evaluation and avoidance of anchoring bias are essential when multiple concurrent pathologies may coexist. Recognition of separate disease processes in this patient allowed appropriate management of both an incidental hepatic hemangioma and a pleural empyema.

## References

[1] Leon M, Chavez L, Surani S (2020). Hepatic hemangioma: what internists need to know. World J Gastroenterol.

[2] Kacała A, Dorochowicz M, Matus I (2024). Hepatic hemangioma: review of imaging and therapeutic strategies. Medicina.

[3] Klotz T, Montoriol PF, Da Ines D, Petitcolin V, Joubert-Zakeyh J, Garcier JM (2013). Hepatic haemangioma: common and uncommon imaging features. Diagn Interv Imaging.

[4] Iguina MM, Danckers M, Sharma S (2024). Thoracic empyema. StatPearls [Internet].

[5] Choi BY, Nguyen MH (2005). The diagnosis and management of benign hepatic tumors. J Clin Gastroenterol.

[6] Bajenaru N, Balaban V, Săvulescu F, Campeanu I, Patrascu T (2015). Hepatic hemangioma -review-. J Med Life.

[7] Ly DP, Shekelle PG, Song Z (2023). Evidence for anchoring bias in physician decision-making. JAMA Intern Med.

[8] Croskerry P (2003). The importance of cognitive errors in diagnosis and strategies to minimize them. Acad Med.

[9] Shen KR, Bribriesco A, Crabtree T (2017). The American Association for Thoracic Surgery consensus guidelines for the management of empyema. J Thorac Cardiovasc Surg.

[10] Heerink WJ, de Bock GH, de Jonge GJ, Groen HJ, Vliegenthart R, Oudkerk M (2017). Complication rates of CT-guided transthoracic lung biopsy: meta-analysis. Eur Radiol.

[11] Rivera MP, Mehta AC, Wahidi MM (2013). Establishing the diagnosis of lung cancer: diagnosis and management guidelines. Chest.

[12] Graber ML, Franklin N, Gordon R (2005). Diagnostic error in internal medicine. Arch Intern Med.

